# In-Bag Dry- vs. Wet-Aged Lamb: Quality, Consumer Acceptability, Oxidative Stability and In Vitro Digestibility

**DOI:** 10.3390/foods10010041

**Published:** 2020-12-25

**Authors:** Renyu Zhang, Michelle J. Y. Yoo, Carolina E. Realini, Maryann Staincliffe, Mustafa M. Farouk

**Affiliations:** 1 School of Science, Faculty of Health and Environment Sciences, Auckland University of Technology, Auckland 1010, New Zealand; renyu.zhang@agresearch.co.nz; 2 Meat Quality Team, Food & Bio-Based Products, AgResearch Ltd., Grasslands, Palmerston North 4442, New Zealand; carolina.realini@agresearch.co.nz (C.E.R.); mustafa.farouk@agresearch.co.nz (M.M.F.); 3 Knowledge & Analytics, AgResearch Ltd., Ruakura Research Centre, Hamilton 3214, New Zealand; maryann.staincliffe@agresearch.co.nz

**Keywords:** in-bag dry-ageing, lamb chops, consumer acceptability, lipid oxidation, protein carbonyl, digestibility, free amino acids

## Abstract

The aim of this study was to produce in-bag dry-aged lamb and compare its meat quality, consumer acceptability, oxidative stability and in vitro digestibility to the wet-aged equivalents. Significantly higher pH, weight loss and reduced cook loss were observed in dry-aged lamb compared to the wet-aged (*p* < 0.0001). Dry-aged lamb had harder and chewier texture profiles and lower colour attributes (L*, a* and b*) than the wet-aged (*p* < 0.001). The dry-aged and wet-aged lamb were equally preferred (around 40% each) by the consumer panel, underpinning the niche nature of dry-aged meat. Significantly (*p* < 0.05) higher yeast and thiobarbituric acid reactive substances (TABRS) levels were observed in dry-aged lamb compared to the wet-aged. There was no difference in fatty acid profile, protein carbonyl content and pattern of proteolysis between ageing regimes (*p* > 0.05). Ageing regimes had no impact on overall digestibility; however, a greater gastric digestibility was observed in dry-aged lamb through the increased release of free amino acids (FAAs) compared to the wet-aged. Outcomes of this study demonstrated for the first time the possibility of producing dry-aged lamb legs of acceptable quality, oxidative stability and superior digestibility compared to the equivalent wet-aged lamb.

## 1. Introduction

Dry-ageing is a processing technique for adding value to meat products. Dehydration, proteolysis, lipolysis and oxidation take place during dry-ageing to produce unique and intensified aged, sweet, brothy, nutty, buttery and roasted flavours compared to the widely practiced wet-aged equivalents [1,2]. Extensive studies have been carried out on various forms of dry-ageing of beef [3,4,5], with comparatively limited research carried out on lamb, although lamb is consumed widely around the world for its nutritional and sensorial qualities. Lamb is a good dietary source of nutrients, including iron, vitamins, high quality protein and polyunsaturated fatty acids (PUFAs), especially eicosapentaenoic acid (20:5, EPA) and docosahexaenoic acid (22:6, DHA), which are the essential omega-3 PUFAs linked to health-promoting functions [6]. Dry-aged beef is mostly produced from the middle cuts, particularly the loin, cube roll and tenderloins [4]. The equivalents of these cuts in lamb are too small to be profitably dry-aged for commercial purposes. The part of a lamb carcass that could be viably used for dry-ageing is the hindleg, known to consumers as lamb/leg chops at retail and when cooked and served in restaurants. Sheep/lamb meat consumers are familiar with wet-aged lamb chops; however, their acceptability towards the dry-aged equivalent remains unknown. 

Many factors contribute to variations in the eating quality of dry-aged meat; some of these include ageing conditions [7], level of moisture evaporation [8] and proliferation of microorganisms [9,10]. Pathways responsible for the development of characteristic dry-aged quality remain to be fully explored. One of the features with dry-ageing is the direct exposure of meat to oxygen in the atmosphere, which could trigger oxidative damage to lipids and proteins, producing oxidative by-products such as lipid/protein-derived carbonyls [11,12,13]. Oxidation has been suggested to modify the protein ultrastructure and result in protein carbonylation and aggregation [14]. Such modifications of proteins may eventually affect the solubility and functionality, leading to a decrease in proteolytic degradation and aggregation during ageing [15] and gastrointestinal digestion [16]. Interactions between oxidative changes arising from the use of different ageing regimes, proteolytic pattern and protein digestibility have not been explored.

Severe oxidative damage to lipids and proteins results in detrimental impacts on the sensorial, nutritional and functional qualities of meat [17,18]. To overcome these issues, dry-ageing in a moisture-permeable ageing bag, so called “in-bag dry-ageing”, has been recently developed. With the use of an ageing bag as a barrier to the atmosphere, improvements in microbiological safety and product yield were also achieved when compared to the traditional out-of-bag dry-ageing [3,19]. Therefore, the aim of the present study was to produce dry-aged lamb legs using water-permeable ageing bags and to compare the dry-aged chops to their wet-aged equivalents in terms of quality, consumer acceptability, oxidative stability and in vitro digestibility. 

## 2. Materials and Methods 

### 2.1. Sample Collection and Ageing Regimes

**Figure 1:**
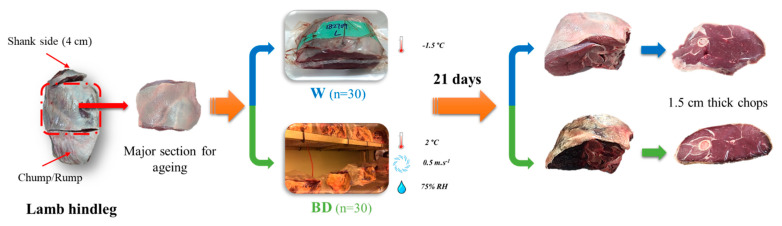
A schematic diagram outlining the ageing process and chops to produce in-bag dry-aged (BD) and wet-aged (W) lamb chops.

Sixty lamb legs were collected from thirty carcasses (Ram, approx. 46 weeks and 26 kg carcass weight, *n* = 60) at a local abattoir on the day of slaughter. Each leg (bone-in, rump muscles on, shank-off) was further portioned into three parts as shown in Figure 1. The main section, after cutting off the rump muscles and 4 cm from the shank side, was used for ageing. The left or right side of lamb leg (from the same animal) was randomly assigned to two ageing regimes: (1) in-bag dry-ageing using water-permeable ageing bag (TUBLIN^®^ 10, 50 μm thick, polyamide mix with water vapor transmission rate 920 g/50 μ/m^2^/24 h at 7 °C, 50% RH, and oxygen transmission rate 660 g/m^2^/24 h at 7 °C, 50% RH, TUB-EX ApS, Denmark) at 2 ± 0.5 °C, 0.5 m·s^−1^ air velocity and relative humidity of 75 ± 5%; (2) wet-ageing in water-impermeable barrier bags (Cryovac^®^ A600 barrier bag, oxygen transmission rate 20–50 g/m^2^/24 h at 23 °C, Sealed Air^®^, Auckland, New Zealand) at −1.5 ± 0.5 °C as the control. The ageing chamber comprised two compartments, as illustrated by Kim [7]: (1) an environmental test chamber (walk-in) as the main chilling chamber with control of temperature, humidity and air velocity; (2) four tunnel chambers located inside the chilling chamber with heating elements at one end and exhaust fan at the other end of the chamber to render more precise control of temperature, humidity and air velocity. Samples of both treatments were aged for 21 days. The sample weight before and after ageing was recorded to calculate the % weight loss from 21 days of ageing. Aged samples from both treatments were fabricated into chops (1.5 cm thick) with no trimming of dry surface for further quality and sensory analyses. The lamb chop for chemical analysis was minced and subsamples were taken from the same chop.

### 2.2. Weight Loss, pH and Proximate Content

#### 2.2.1. Ageing Weight Loss (%)

Sample weight was recorded at an interval of three days to calculate % weight loss from ageing: % Ageing weight loss = ((Initial weight of sample before ageing−Weight at a given time point)/Initial weight before ageing) × 100. A correlation study between the initial sample weight and the weight after 21 days of ageing was also carried out to estimate the yield of dry-aged lamb under the current processing conditions based on the initial weight. 

#### 2.2.2. Cook Loss and Percentage Total Loss (%)

Dry- and wet-aged lamb chops were vacuum packaged and cooked sous vide in vacuum barrier bags at 72 °C for 1 h, then cooled in an ice bath for 1 h before they were transferred to 0 °C chiller overnight. The sample weight before and after cooking were recorded to calculate the percentage of cook loss. The percentage of total loss was calculated by combining the percentage of ageing loss and the percentage of cook loss.

#### 2.2.3. pH

pH values of lamb samples before and after the ageing treatments were measured by inserting a calibrated pH probe (Hanna 99,163 pH meter with a FC232D combined temperature and pH insertion probe, Woonsocket, RI, USA) directly into the meat. The calibration of pH meter was carried out with 2-point standard buffer solution of 7.01 and 4.01 at ambient temperature. Measurement was carried out in duplicate for each sample. 

#### 2.2.4. Proximate Content

The lamb chop for proximate analysis was minced and subsamples were taken from the same chop (*n* = 60). Moisture and crude fat content were determined using standard methods of AOAC 950.46 and AOAC 960.39, respectively [20]. The total muscle proteins were extracted following the method described by Zhang [5] using total muscle extraction buffer (50 mM Tris-HCl, pH 5.8; 10% glycerol; 2% SDS and 2% 2-mercaptoethanol). The concentration of protein solution was determined using RC-DC protein assay kit (Bio-Rad^®^ Laboratories, Hercules, CA, USA). 

### 2.3. Surface Microorganism Enumeration

Approximately 10 g of samples were excised from untrimmed meat surface (2 mm thick) post-ageing, using a boner’s knife sanitised in 70% alcohol. Samples were collected in sterile Whirlpak bags (Nasco, Madison, WI, USA), and transported on ice to the laboratory. Meat samples were placed into a stomacher bag and homogenised with 100 mL maximum recovery diluent (MRD, Difco, Detroit, MI, USA), with subsequent serial dilutions. Enumeration of microorganisms growing on the untrimmed surface of dry-aged lamb was determined using standard methods as described in the Compendium of Methods of Microbiological Examination of Foods [21] for *Escherichia coli* (*E. coli*, Chapter 8.91, equivalent to ISO 7251:2005) using the multiple tube technique in Lauryl Tryptose broth for 24 h at 44 °C; aerobic plate count (Chapter 7.62, equivalent to ISO 4883-2:2013) using standard Plate Count Agar incubated for 72 h at 35 °C; lactic acid bacteria (Chapter 19.522, equivalent to ISO 15214:2000) using de Man, Rogosa and Sharpe agar incubated for 72 h at 30 °C; *Enterobacteriaceae* (Chapter 8.63, equivalent to ISO 21528-2:2004) using Violet Red Bile Glucose agar for 24 h at 37 °C; mould and yeast (Chapter 20.51, equivalent to ISO 21527-1:2008) using Dichloran Rose-Bengal Chloramphenicol Agar for 5 days at 25 °C. Results for *E coli* were expressed as log most probable number (MPN)/g meat, and results for other microbial analysis were expressed as log colony-forming unit (cfu)/g meat.

### 2.4. Instrumental Colour

Freshly cut lamb chops from dry- or wet-ageing were placed in a polypropylene foam tray with absorbent meat pad and overwrapped with polyvinyl chloride film and allowed to bloom at 4 °C for 1 h. Instrumental colour was measured on the surface of four major muscles, *m. semimembranosus* (SM), *m. biceps femoris* (BF), *m. vastus lateralis* (VL) and *m. rectus femoris* (RF), using a calibrated Minolta Chroma Meter (CR-400; Konica Minolta Photo Imaging Inc., Mahwah, NJ, USA). Three random positions were measured on each muscle. The colour coordinates of CIE L* (lightness), a* (redness) and b* (yellowness) were measured using Illuminant D65 with 8 mm diameter aperture. Chroma and hue angle were further calculated to describe the colour properties of the samples according to Zhang [22]. 

### 2.5. Instrumental Texture Profile Analysis

Texture profile analysis was performed on four major muscles (SM, BF, VL and RF) of the cooked lamb chops from Section 2.2.2. Compression test was carried out using Stable Micro System TA.HD Plus texture analyser (Surry, UK) with a maximum loading force of 50 kg. Each muscle was cut into 1 cm^3^ cubes (minimum 6 cubes per muscle) and measured against the grain using a 50 mm cylinder probe. The compression test was performed at 50% strain with the test speed of 5.0 mm·s^−1^ and trigger force of 5 g. At least 6 measurements were taken for each muscle.

### 2.6. Consumer Sensory Evaluation 

Lamb chops were sous vide cooked in vacuum barrier bags at 65 °C for 75 min and then grilled for 60 s each side at approx. 180 °C to obtain a core temperature of 72 °C. The samples were then cut across the muscles to obtain two slices per chop. Each consumer was served with two slices of lamb, both slices were from the same carcass but different ageing regimes. The lamb slices were placed on a plate which was coded with two randomly selected IDs for dry- or wet-aged lamb. Consumers were informed that the two samples were from different ageing regimes and served in a random order. A group of 114 high-income (≥NZD 70,000/year) consumers who could afford dry-aged products participated in the consumer sensory evaluation (one session). Panellists were asked to rate the degree of liking using a 9-point hedonic scale (1 = dislike extremely to 9 = like extremely) and rate the eating quality using a 5-point hedonic scale (1 = unsatisfactory as an everyday product, 2 = good everyday product, 3 = slightly better than an everyday product, 4 = almost a premium product and 5 = a premium product). The procedures used for consumer sensory evaluation in this study have been approved by the Auckland University of Technology Ethics Committee.

A focus group is recommended to generate perceptions and/or hypotheses for an area when previous knowledge about the area is limited [23,24]. A six-member focus group was organised as a complementary study to explore the sensory descriptors of in-bag dry-aged lamb. A group of 12 persons was selected from the consumer sensory panel who participated in the acceptability evaluation of dry-aged lamb, and then they were subjected to further screening. They were screened according to the following criteria: (1) high income; (2) age range of 25 to 70 years; (3) high level of education (degree or higher), (4) confident to verbally express their opinions (from the 12, we selected 6 that voiced a strong positive or negative opinion on the product). The focus group discussion followed a semi-structured interview protocol according to Rabiee [25], which is summarised in Table 1.

The focus group was directed by three people, which comprised one moderator and two assistants. The moderator facilitated the discussion and the assistants took notes. The session lasted approximately 60 min, and the discussion was recoded on an audio recorder. All the participants were seated together in a relaxed environment at home to mimic the type of environment where the product is likely to be consumed. The lamb was cooked (the same cooking method as the consumer study) in the kitchen and then presented on a table with other food accompaniments (e.g., sauces, breads and vegetables). The participants were invited to serve themselves as much lamb and other items that they wanted. They were able to serve themselves more lamb at any time during the session. Participants were informed that there were no right or wrong answers to the questions and that we were interested in gaining a better understanding of their perceptions of the product. They were encouraged to freely express their attitudes regarding the product.

### 2.7. Protein Carbonyl Content 

Whole muscle protein of lamb samples was extracted using the method as described in Section 2.2.4. Concentrations of protein carbonyl groups generated from protein oxidation were measured using the 2,4-dinitrophenylhydrazine (DNPH) method proposed by Levine [26]. The extracted total muscle proteins were used and the protein extraction buffer served as a blank to react with DNPH. Absorbance of carbonyl solution was read at 370 nm using a UV-spectrophotometer after suspending in 6 M guanidine HCl (in 20 mM sodium phosphate buffer, pH = 2.3) against the blank. The measurement was performed in duplicate.

### 2.8. Lipid Oxidation and Fatty Acid (FA) Profile

Extent of lipid oxidation was determined based on the content of thiobarbituric acid reactive substances (TBARS) according to Buege [27]. FA profile was determined according to Zhang [22].

### 2.9. In Vitro Digestion of Lamb Chops

In vitro digestion of dry- and wet-aged lamb chops was performed using a static enzymatic digestion method modified from Zhang [22]. A two-stage digestion of 240 min (120 min of gastric digestion and 120 min of intestinal/pancreatic digestion) was simulated in a bioreactor in a shaking water bath (Thermo Haake DC 10, Karlsruhe, Germany) set at 37 ± 0.2 °C and 80 rpm. Simulated solutions of Gastric Fluid (SGF, pH = 3) and Intestinal Fluid (SIF, pH = 7) were prepared with pepsin (P6887, Sigma, Auckland, New Zealand) and pancreatin (ACROS Organics^TM^, Thermo Fisher Scientific), respectively. Lamb chops were minced and approximately 4 g (with same protein content) of sub-sample was homogenised with 5 mL of simulated salivary fluid (pH = 7) at 22,000 rpm (IKA Labortechnik, Germany) for 20 s, twice to simulate mastication. Pepsin solution was added to the bioreactor to initiate gastric proteolysis (enzyme to substrate ratio of 1:278, pH = 2) [28]. At 0, 2, 10, 60 and 120 min of gastric digestion, two aliquots of 250 µL hydrolysates were removed and immediately mixed with either methanol (1:2, *v/v*) for free amino acid (FAA) analysis or SDS-PAGE sample loading buffer (1:1, *v/v*, 50 mM Tris-HCl, pH 6.8; 10% glycerol; 2% SDS; 5% 2-mercaptoethanol and 0.02% bromophenol blue) for SDS-PAGE gel electrophoresis. Aliquots for gel electrophoresis were heated at 95 °C for 5 min and then stored at −80 °C until further analysis. Pancreatin solution of enzyme to substrate ratio of 1:100 was added to initiate the intestinal digestion at pH 7 for 120 min. Aliquots of hydrolysates were removed from the reactor and treated the same as those collected in the gastric phase. 

#### 2.9.1. SDS-PAGE Gel Electrophoresis

Hydrolysates collected from the digestion simulation were loaded onto Novex™ NuPAGE™ 10% Bis-Tris Midi Protein Gels (Invitrogen^TM^, Thermo Fisher Scientific, Auckland, New Zealand) by 40 µg proteins per well and separated at ambient temperature in a Bio-Rad Criterion cell system at 150 V using PowerPac™ HC High-Current Power Supply (Bio-Rad^®^ Laboratories, Hercules, CA, USA). An 8-µl aliquot of Novex™ Sharp Pre-stained protein standard (Invitrogen, UK) was used to determine molecular weight (MW) of different protein sizes from 3.5 to 260 kDa. Following electrophoresis, gels were stained in a SimplyBlue SafeStain (Invitrogen^TM^) for 4 h. Stained gels were then washed with distilled water and images were captured with a GS900 calibrated densitometer scanner (Bio-Rad^®^ Laboratories). The gels were loaded following the order which enabled the time-course of digestion (0, 2, 10, 60, 120 and 240 min) to be visualised and for dry- and wet-ageing treatments to be compared.

#### 2.9.2. Analysis of Free Amino Acids (FAAs)

Aliquots for FAA profile were centrifuged at 10,000× *g* for 10 min. The supernatant containing FAA extract was quantified by Agilent 1260 Infinity HPLC system equipped with Agilent 6420 Triple Quadrupole LC/MS system (Agilent Technologies New Zealand Limited, Wellington, New Zealand), according to Zhang [29]. The AA standard solution (100 µM) of 40 amines (included internal standard) prepared with the 37 AA standard mixture (A9906, Sigma, Auckland, New Zealand), asparagine (A0884, Sigma, Auckland, New Zealand) and glutamine (G3126, Sigma, Auckland, New Zealand) was serially diluted to 0.78 µM to generate a standard curve for the identification and quantification of FAAs using MassHunter software (Agilent Technologies). The final concentration of FAAs was expressed as mg/g protein.

#### 2.9.3. Relative Protein Digestibility (%)

The relative protein digestibility following 240 min of simulated gastrointestinal digestion was determined by three methods: (1) the protein profile using SDS-PAGE electrophoresis (Section 2.9.1), (2) the release of FAAs (Section 2.9.2) and (3) the protein content (protein fragments and peptides) in the hydrolysates after pancreatic digestion using RC-DC protein assay kit (Bio-Rad^®^ Laboratories, Hercules, CA, USA). Relative digestibility (%) was calculated as follows:

(1) Relative digestibility _SDS-PAGE_ = (1 − (Optical intensity of protein bands <10 kDa/Optical intensity of all protein bands)) × 100
(2) Relative digestibility _FAAs_ = ((Total FAAs (g) at 240 min − total FAAs (g) at 0 min)/Protein content of the sample) × 100
(3) Relative digestibility _Protein content_ = (1 − Protein content (g) in hydrolysate at 240 min/Protein content of the sample) × 100

### 2.10. Statistical Analysis

A randomised control trial was designed with thirty pairs of lamb legs (*n* = 60) which were evenly assigned to two different treatments: in-bag dry-ageing and wet-ageing. A model including the fixed effect of ageing treatments and the random effect of carcass ID and carcass sides was used for chemical analysis of minced chops. For instrumental colour and texture profile analysis, the ageing treatments and muscles were considered as fixed effects, and carcass ID and carcass sides were considered as random effects fitted in the model. For the sensory evaluation analysis, the ageing treatments were included as the fixed effects; panellists, carcass ID and muscles of lamb chops were included as the random effect in this model. Linear mixed effect regression analyses were performed on the data using R (version 3.4.1), with “lme4” and “predictmeans” packages to determine the difference between ageing treatments. Analysis of variance (ANOVA, one-way) was used to investigate the effect of different ageing treatments with a post-hoc comparison of means performed using Fisher’s least significant differences (LSD) and Tukey’s (HSD) test at 5% significance level. A power curve was generated to describe the relationship between % ageing weight loss and ageing time under the current dry-ageing process. The Chi-squared test was performed on the eating quality rating of two ageing methods at 5% significance level. 

## 3. Results and Discussion

### 3.1. Meat Quality

#### 3.1.1. Weight Losses

**Figure 2:**
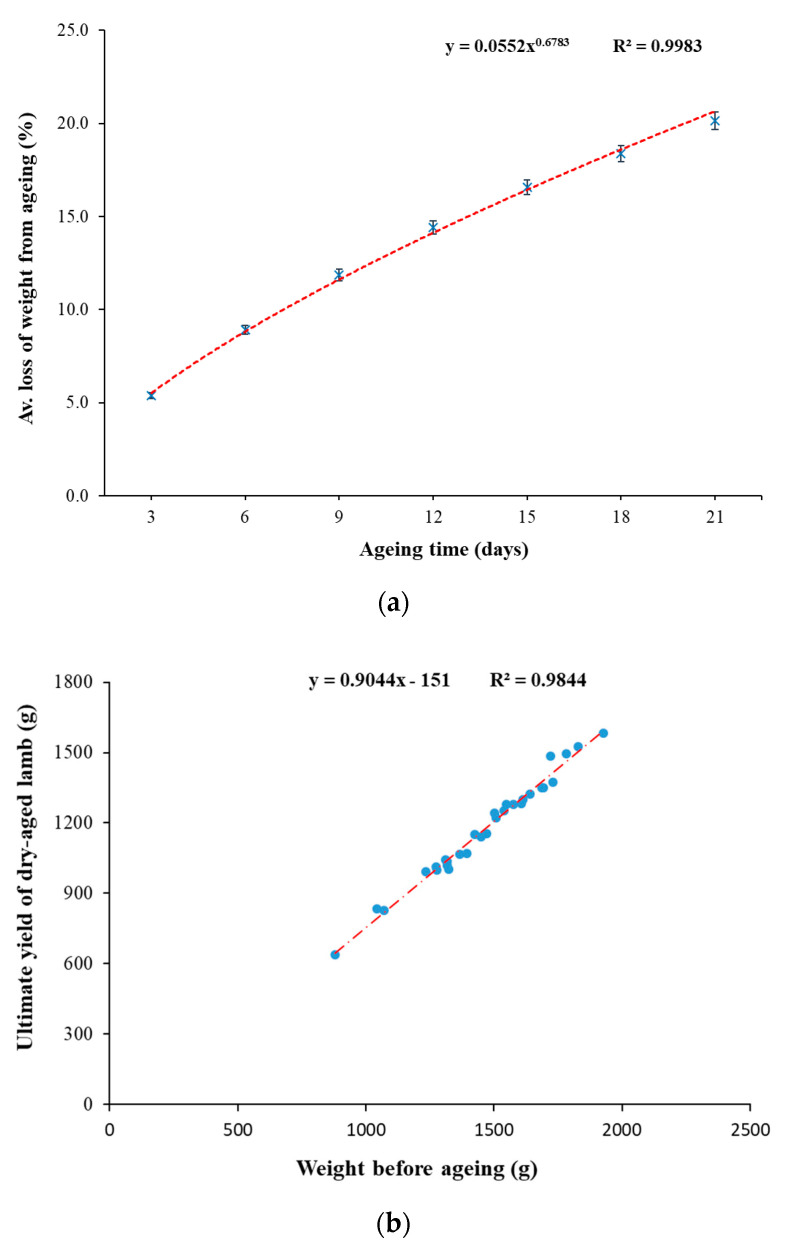
(**a**) Average loss of weight from ageing (%) observed in in-bag dry-aged lamb across 21 days of ageing time. A trendline was added to establish the power relationship between the weight loss from ageing with ageing time. Error bars represent standard errors (*n* = 30). (**b**) Initial weight (g) of lamb legs before ageing and the ultimate yield (g) of in-bag dry-aged lamb (*n* = 30). A trendline was added to predict the linear relationship between the weight of lamb samples before and post in-bag dry-ageing.

The weight losses during ageing and cooking were measured to estimate the yield of dry-aged lamb chops from processing to the point of consumption. In-bag dry-ageing resulted in a significantly (*p* < 0.0001) higher weight loss than wet-ageing, which was expected in dry-aged meat products. The change in weight loss from dry-ageing over time is shown in Figure 2a. In general, around 20% of moisture was lost from dry-aged lamb after 21 days of ageing time as compared to the control with an average of 0.71% purge loss. The relationship (*R*^2^ = 0.9983) between % weight loss from ageing and ageing time under the current dry-ageing process is shown as follows:

Y = 0.0552X^0.6783^ × 100

Y = % weight loss from ageing;
X = Days of ageing

The correlation between the initial weight (applicable range: 0.5–2 kg) and dry-aged weight (21 days) was calculated to predict the yield of dry-aged lamb when the initial weight was known (Figure 2b). A close to linear relationship (*R*^2^ = 0.9844) was found as follows:

Y = 0.9044X − 151

Y = Dry-aged weight (g) at 21 days of ageing;
X = Initial weight (g) before ageing

Cook loss of dry-aged lamb was significantly reduced to an average value of 16.94% compared to the control (27.79%, Table 2). Similar findings were reported on beef products where cook loss was lower in the dry-aged beef than wet-aged counterparts [19,30,31]. Although the total loss (ageing weight loss + cook loss) remained significantly higher in dry-aged samples (36.52%) than the control (28.48%), the difference between the two ageing treatments was reduced to less than 10%.

#### 3.1.2. Proximate Content and pH

The moisture content of dry-aged lamb (67.30%) was significantly (*p* < 0.05) lower than the wet-aged counterparts (73.25%), which contradicts the outcomes of other studies that found no difference in moisture content between wet- and dry-ageing treatments [3,30]. This could be attributed to dehydration as part of the dry-ageing process mainly occurring on the outer surface of the meat. Most studies have trimmed the surface crust of dry-aged meat prior to sampling for moisture content analysis, thus eliminating the difference that would have been observed [5]. With the use of dry-ageing bags, no trimming was necessary in this study. Lower moisture levels in dry-aged lamb were expected owing to the significantly higher weight loss in the in-bag dry-aged sample compared to wet-aged after 21 days of ageing. 

Dry-aged lamb had higher (*p* = 0.058) crude fat (6.82%) and protein (20.22%) contents compared to the wet-aged control (5.93% fat and 16.54% protein), which agreed with the outcomes of other studies on beef [30,31]. The increase in fat and protein content in the current study may be attributed to the significant decrease in moisture content, which concentrated the other components of lamb.

Both ageing methods significantly (*p* < 0.05) increased the pH values of lamb from the average value of 5.83 (before ageing) to 5.92 (wet-aged) and 6.04 (dry-aged), respectively (Table 2). The increase in pH after ageing has been well reported on beef [5,32,33], which could be associated with the production of nitrogenous compounds by proteolysis. (In-bag) dry-ageing has been reported to increase the pH of beef loins [19] with ageing time and for the dry-aged samples to have a higher pH value than the wet-aged counterparts [1]. 

#### 3.1.3. Surface Microorganism Growth

Overall, the surface microorganism counts were low in both dry- and wet-aged lamb (Table 2). A significantly (*p* < 0.05) higher level of yeast was found in dry-aged lamb with the lower aerobic bacterial counts compared to the wet-aged. It is speculated that the low moisture environment on the surface of dry-aged meat favoured the proliferation of yeast, which consumed the available oxygen on the meat’s surface and consequently outcompeted the aerobic bacteria. There remains a need for future study to confirm the current hypothesis. A higher yeast level was also observed in dry-aged beef than the wet-aged counterparts [1,5,19]. There were no *E. coli* and moulds detected in both treatments, which was also observed in a previous study on beef [34]. Lactic acid bacteria and *Enterobacteriaceae* counts were found higher on the surface of wet-aged lamb samples than the dry-aged. Similar findings of lactic acid bacteria were also reported by Li [19] between wet- and dry-aged beef, which could be attributed to the anaerobic environment of wet-ageing favouring the proliferation of lactic acid bacteria. Contradictory results have been reported on the *Enterobacteriaceae* counts of dry-aged beef being higher [1] or similar [19] compared to the wet-aged control. 

#### 3.1.4. Instrumental Colour

The ageing process affected the colour properties of lamb. All the colour attributes were lower (*p* < 0.05) in dry-aged lamb than the wet-aged except for hue angle and VL a* (*p* > 0.05) (Table 3). Overall, in-bag dry-ageing generated a slightly darker, less red and less yellow colour, which could be associated with the moisture loss during the ageing process. The dehydration of dry-aged lamb reduced the light reflection and concentrated colour components including myoglobin and iron that result in changes in the meat colour. Similar effects of dry-ageing on beef colour were reported by Kim [7]. Colour plays an essential role in consumer acceptability of lamb. The minimum thresholds for L* and a* for consumer colour acceptability have been reported as 34 to 35 and 9.5, respectively [35]. The colour of the lamb samples from both ageing treatments were within the acceptable colour range; thus, in-bag dry-ageing had no negative effect on the colour quality of lamb after 21 days of ageing. 

There was no difference in the way in which the ageing treatments affected the colour of the four muscles of lamb chops, though an exception was seen for the VL muscle of dry-aged lamb, which was less brown (lower hue) than the wet-aged control (*p* < 0.05, Table 3). The impact of different muscles on the colour properties in the current study may be associated with the inherent variations across muscles, including pH, the content of fat, iron and myoglobin [36] and also the oxidative stability of myoglobin [37].

#### 3.1.5. Instrumental Texture Profile Analysis

Most of the study on dry-aged beef products focused on the tenderness of the meat as compared to the wet-aged. However, the texture profile of dry-aged products has not been well explored, particularly for dry-aged lamb. The majority of studies on beef failed to detect any significant difference in shear force between dry- and wet-aged beef [7,19,31,38], suggesting that improvement in tenderness was not an advantage of dry-aged products over wet-aged, considering that both ageing methods can effectively tenderise the meat to similar levels. 

As shown in Table 4, both ageing treatments produced lamb samples which were tender and easy to chew (low force values for hardness and chewiness). Significantly (*p* < 0.05) higher hardness and chewiness were detected in dry-aged lamb compared to wet-aged, likely as a result of dehydration of the samples, which caused shrinkage and resulted in a firm texture. Cohesiveness and resilience of dry-aged lamb were slightly (*p* < 0.05) lower as compared to the wet-aged control. However, the difference between the two treatments was numerically too small to have a significant impact on the textural properties of the meat.

Lamb legs consist of multiple muscles. Different muscles were affected differently by the ageing treatments. As shown in Table 4, the type of muscle only influenced the hardness, chewiness and springiness of lamb samples. A significant (*p* < 0.05) difference in hardness between ageing treatments was seen in SM and VL muscles. Dry-aged lamb was slightly chewier than the control; however, a significant (*p* < 0.05) increase was only observed in BF and VL muscles. 

### 3.2. Sensory Quality

#### 3.2.1. Consumer Acceptability

The palatability of in-bag dry-aged lamb has not been previously evaluated. As shown in Table 5, both lamb samples were equally preferred (*p* > 0.05) by the consumer panellists as a “better than everyday product”. The average degree of preference for both samples (in-bag dry-aged = 6.68, wet-aged = 6.75) was close to “like moderately”, which was score 7. 

The sensory quality of in-bag dry-aged beef compared to wet-aged has been extensively studied but the results were inconsistent. For instance, in-bag dry-aged beef samples have been reported to be preferred by consumers to the wet-aged counterparts [7,19,39]; no difference [40,41]; or wet-aged products preferred [38]. The conflicting findings may have arisen from the use of different ageing processes, types of muscles and breeds. 

Dry-aged meat is a niche product which is expected to be only preferred by a certain group of consumers. Therefore, the debate on different ageing methods should be focused on how many (more) consumers would prefer dry-/wet-aged products instead of which products were more palatable than the other because they are all palatable. In the current study, 44.74% of consumers rated wet-aged lamb higher in terms of overall preference. This was more likely owing to their familiarity with wet-aged lamb products. It was promising to observe a similar number (40.35%) of consumers that preferred in-bag dry-aged lamb to the wet-aged counterparts (44.74%). There was only a small group (14.95% on overall preference) of consumers who could not distinguish between in-bag dry-aged and wet-aged lamb. In eating quality rating, there was no significant difference between the average score of in-bag dry-aged and wet-aged lamb. Both in-bag dry- and wet-aged samples were considered by most of the consumers (71.68% and 68.14%, respectively) as slightly higher than an everyday product (score 3). Considering that these were lamb chops and not lamb loins that were assessed, this overall level of acceptability is highly promising for this lamb cut. No significant difference (chi-square; *p* = 0.670) was found in the distribution of response along the 5-point scale consumer rating of eating quality between the two ageing methods (Table 5). Therefore, the current findings suggest a niche market for dry-aged products and a great market potential for in-bag dry-aged lamb.

#### 3.2.2. Focus Group Perceptions of In-Bag Dry-Aged Lamb Chops

In the present study, a focus group was used phenomenologically to strengthen the outcomes of the quantitative survey with the targeted consumers due to the paucity of information on dry-aged lamb. Members of the focus group in the present study discussed and described the characteristics of in-bag dry-aged lamb chops based on their eating experience of lamb products. Out of the six members, only one member did not like the in-bag dry-aged lamb chop because of the drier texture, though the same person perceived the flavour of the meat as pleasant. 

Appearance and aroma: The “lean (not fatty)” appearance of in-bag dry-aged lamb chops was considered as an advantage. “Dry (not juicy)” appearance was another attribute of in-bag dry-aged lamb which may be one of the key features of dry-aged products. The dehydration process of dry-ageing caused a certain level of moisture loss and resulted in a “dry look”. All the focus group panellists strongly liked the (cooked meat) aroma of the in-bag dry-aged lamb and described it as “fine/pleasant aroma” and “less mutton smell (unpleasant) than the normal lamb”. 

Texture: All of the group members agreed that the texture of in-bag dry-aged lamb was “tender”, “spongy” and “less fibrous”. These attributes were considered as positive descriptors for the texture characteristics of in-bag dry-aged lamb chops. These attributes were also observed from the consumer sensory session, which found that the consumers who preferred in-bag dry-aged lamb liked the “chewier” texture rather than too soft/tender texture of the wet-aged lamb.

Taste/flavour: The taste/flavour of in-bag dry-aged lamb was considered as nutty, sweet with aftertaste, venison-like (gamey), stronger lamb flavour (pleasant) but no mutton flavour (unpleasant), no fatty taste or greasy aftertaste (even when cold). A stronger umami and sweet taste, buttery, nutty, brothy and roasted flavours were also detected previously in (in-bag) dry-aged beef samples as compared to the wet-aged counterparts [1,42]. The suggestion by the focus group that dry-aged lamb left no greasy coating is highly significant as one of the issues that consumers have with lamb meat is the coating of the mouth and throat often experienced on eating lamb due to the high melting temperatures of lamb fat [43,44]. This quality change as a result of dry-ageing needs to be further ascertained and, if confirmed, should be used to differentiate dry-aged lamb from its wet-aged equivalent. 

### 3.3. Protein and Lipid Oxidation 

#### 3.3.1. Protein Carbonyl Content 

Assessment of protein carbonyl content has been widely used to estimate the extent of protein oxidation. As shown in Table 6, overall, the protein carbonyl content was low in both lamb samples (*p* > 0.05). The protein carbonyl naturally exists in the animal tissues at a level of 1–2 nmol.mg^−1^ and increases during rigor mortis [11,13]. It has been reported that post-mortem ageing may promote the carbonylation of meat proteins [13,29]. However, the influence was highly dependent on the source of meat, type of muscle and, particularly, the storage conditions [45]. Storage under high-oxygen atmosphere at higher temperature or exposure to the reactive agents such as light, transition metals (Fe^3+^/Cu^2+^) and oxidising lipids could also trigger protein oxidation. In the current study, the barrier function of the ageing bag limited the oxygen availability in the meat [19], which reduced the potential for the carbonylation of protein and resulted in a similar carbonyl level as the control. 

#### 3.3.2. TBARS and FA Profile

Lipid is more susceptible to oxidative damage than protein during post-mortem storage [12]. TBARS has been widely used to evaluate the extent of lipid oxidation by measuring the content of lipid peroxidation products, malondiadehyde (MDA), present in the samples [46]. 

As shown in Table 6, a significantly higher TBARS value was detected in dry-aged lamb compared to the wet-aged. However, the TBARS level in the current study was below the rancidity threshold of 2.0 mg MDA [47]. Jiang [48] found no significant difference in TBARS between dry- and wet-aged beef samples. In-bag dry-ageing was reported to lower lipid oxidation (lower TBARS) in beef products compared to the traditional dry-ageing [30]. As described earlier, dry-ageing is an aerobic maturation process involving a certain degree of oxidation and resulting in oxidative by-products. These oxidative products participate later in cooking and produce a range of volatiles such as aldehydes, hydrocarbons, ketones and lactones, which may contribute towards the flavour intensity of meat [49,50,51].

The FA content was not affected (*p* > 0.05, Table 6) by the ageing treatments, suggesting that the in-bag dry-ageing did not negatively affect the nutritional value of FAs in lamb. It is worth noting that in-bag dry-ageing did not adversely affect PUFAs, including EPA and DHA. Therefore, the nutritional value of lamb was not affected by the current in-bag dry-ageing process in terms of FA profile, although an increase in TBARS level in the in-bag dry-aged lamb was observed. The increase in oxidative potential (TBARS), on the other hand, may be associated with the flavour development of dry-aged products as discussed above. 

### 3.4. Proteolysis

**Figure 3:**
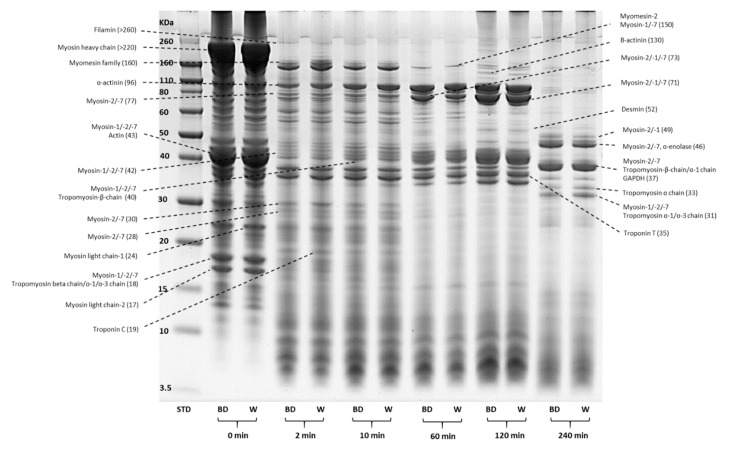
SDS-PAGE protein profile of in vitro gastrointestinal digestion of in-bag dry-aged (BD) and wet-aged (W) lamb chops; protein identification was according to Farouk [28] and Wu [54].

Protein profiles (SDS-PAGE) of in-bag dry- and wet-aged lamb are shown in Figure 3 (0 min). No difference in protein profiles between the ageing regimes was observed, suggesting that a similar proteolytic pattern occurred due to the activities of endogenous proteases regardless of the ageing regimes. Similar protein profile (SDS-PAGE) was detected previously in beef muscles aged by traditional out-of-bag dry-ageing [52], in-bag dry-ageing [29] and wet-ageing regimes. Peptides of smaller than < 3 kDa were present in higher amounts in dry-aged beef compared to the wet-aged [52]. This could be due to the proliferation of microorganisms (mould and yeast) during dry-ageing, which contributed towards the proteolytic activity of peptidases and resulted in different compositions of FAAs compared to the wet-aged [8,10]. In this study, the release of FAAs following in-bag dry-ageing was more evident than that of wet-ageing, where significant (*p* < 0.05) increases were only detected in isoleucine, lysine, aspartic acid and proline (Table 7, 0 min). Such changes in FAA compositions could be explained by two mechanisms: the activity of aminopeptidases due to the growth of yeast (Table 2) and a higher ageing temperature used for the in-bag dry-ageing process. The activity of aminopeptidases by yeast is known to contribute towards the release of FAAs in dry cured/fermented meat products as well [53]. On the other hand, different ageing temperatures (2 vs. −1.5 °C) used in the current study may also have contributed to the changes in the FAA profile between two ageing regimes. A significantly higher level of tryptophan, phenylalanine, valine, tyrosine, glutamate, isoleucine and leucine was detected in dry-aged beef at 3 °C compared to the wet-aged equivalents at 1 °C by Kim [7]. However, such a contributory effect of ageing temperature on the activity of proteases was not supported by the findings of the protein profile between the two ageing regimes in this study. Thus, there remains a need for future study to assess the actual impact of the slight increase in ageing temperature on the proteolytic pattens of in-bag dry-aged lamb.

### 3.5. In Vitro Digestibility 

In vitro protein digestibility was measured in three ways, as described by Zhang [29]: (1) by using SDS-PAGE to determine the changes in protein profiles with four hours of digestion time, simulating peptic and pancreatic digestions in the upper and lower gastrointestinal tract; (2) by measuring the changes in FAAs with the digestion simulation; and (3) by measuring the protein content in the final hydrolysate. 

#### 3.5.1. Changes in Protein Profiles (SDS-PAGE)

**Figure 4:**
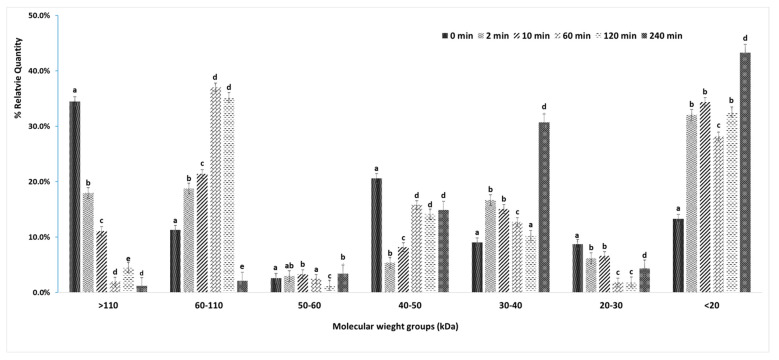
Relative quantity changes (mean ± standard error) of seven major molecular weight protein groups across the gastrointestinal digestion process (0, 2, 10, 60, 120 and 240 min.). The relative quantity of protein fragments significantly differed across the digestion process when means showed different letters “a, b, c, d, e” within the same molecular weight group (*p* < 0.05).

The representative SDS-PAGE protein profiles of in-bag dry- and wet-aged lamb at five sampling time points (0, 2, 10, 60, 120 and 240 min) during four hours of simulated digestion process are shown in Figure 3. The % relative quantity [(% RQ = optical density of the protein fragments/optical density of total proteins) × 100%] was used to compare the quantitative differences of protein fragments between the ageing treatments. Protein fragments of different MW were grouped into seven MW groups (>110, 60–110, 50–60, 40–50, 30–40, 20–30 and <20 kDa) for statistical analysis, as shown in Figure 4. There was no significant (*p* > 0.05) difference in the % RQ of the seven MW groups between in-bag dry- and wet-aged lamb over the digestion period (data not shown). Therefore, statistical analysis of these protein groups was performed on the average % RQ of the two ageing treatments. 

Most of the large-sized proteins, including filamin, titin and nebulin (>250 kDa) and myosin heavy chain (MHC, >220 kDa), were digested rapidly by pepsin and the protein bands disappeared within 10 min of pepsin digestion (Figure 3) [28,54]. Myomesin and myosin family proteins of MW of 150–160 kDa were progressively cleaved through the gastric digestion with accumulation of lower MW (60–110 kDa) protein fragments. This was also seen with the % RQ results, where large MW proteins (>110 KDa) were significantly (*p* < 0.05) reduced over the gastric digestion process with significantly (*p* < 0.05) increased protein fragments of MW between 60 and 110 kDa (Figure 4). 

Protein fragments of MW 50–60 kDa were quickly digested within the first 2 min of gastric digestion, with continuous cleavage throughout the digestion period (Figure 3). Another group of protein fragments with significant changes in protein profiles ranged from 30 to 50 kDa, which mainly consisted of myosin family, actin and tropomyosin family [28,54]. These protein fragments underwent rapid partial digestion by pepsin at the initial stage (2 min). The broken fragments became resistant to pepsin and accumulated throughout the gastric digestion, as evidenced by the significant decrease in the % RQ of proteins (40–50 kDa) within the first 10 min, with no further changes afterward (Figure 4). The low MW protein fragments (10–30 kDa) included myosin-1, -2 and -7, myosin light chain-1 and -2, Troponin C, which were readily digested within 60 min of gastric digestion. The % RQ of low MW proteins (20–30 kDa) significantly reduced over the gastric digestion, while the smaller protein fragments (<20 kDa) increased significantly (*p* < 0.05) within 2 min and then varied slightly throughout the pepsin digestion time of 120 min. 

The protein fragments that resisted gastric digestions (30–50 kDa) were further hydrolysed to smaller fragments during the subsequent 120 min of pancreatic digestion (Figure 3). These fragments may include proline-containing peptides that are reported to resist digestion by pancreatic enzymes [55]. This is further supported by the relatively unchanged amount of proline in the hydrolysates over the digestion simulation period (Table 7). Farouk [28] also observed the resistance of these protein fragments to pancreatic digestion in beef samples. The % RQ of proteins above 60 kDa significantly decreased (*p* < 0.05) at the end of pancreatic digestion (240 min), with a significant (*p* < 0.05) increase in the % RQs of 30–40 kDa proteins. 

#### 3.5.2. Changes in FAAs

The release of FAAs over the four hours of gastrointestinal digestion is shown in Table 7. The FAA level increased to around three times by the end of pancreatic digestion (240 min) compared to the gastric phase levels (120 min). The percentage of EAAs in the total AAs (% EAAs) decreased in the first 2 min of digestion, then remained unchanged throughout the gastric digestion process. However, the increase in % EAA was observed at the end of the gastrointestinal digestion simulation. Current findings indicated that the main role of gastric enzymes (pepsin) was to break down large proteins into smaller fragments ready for more complete digestion by pancreatic enzymes and the concomitant release of FAAs. Pepsin is an endopeptidase which plays a role in the cleavage of peptide bonds within molecules in the P1 and P1’ positions and break down large protein molecules into smaller peptides. The pancreatic enzymes, including endopeptidases (trypsin, chymotrypsin and elastase), exopeptidases (carboxypeptidase A and B) and other proteases, are the major sources of proteases in the human digestive system. It is well known that pancreatic enzymes contribute more (around 40%) than pepsin (10–20%) to proteolysis during human gastrointestinal digestion [56]. 

A significant increase in EAAs at the end of digestion was observed. These consisted of leucine, lysine, phenylalanine, tyrosine and arginine, as previously observed in dry-cured ovine meat [22]. This can be explained by the selective cleavage of peptide bonds, which link the carboxyl side of basic AAs (e.g., lysine and arginine) by trypsin [56], and the preferential cleavage of the linkage between aromatic AAs (e.g., phenylalanine and tyrosine) by chymotrypsin [55]. Elastase is another pancreatic endopeptidase responsible for the cleavage of the carboxyl side of aliphatic AAs, which includes alanine, glycine, isoleucine, leucine and valine [55]. The exposure of these AAs can act as substrates to be further digested by exopeptidases (Carboxypeptidase A and B), which may explain the significant increase in these AAs after 120 min of pancreatic digestion observed in the current study (Table 7).

There was no significant difference between the ageing treatments within the first 10 min of gastric digestion, except for a significantly (*p* < 0.05) higher content of cysteine in dry-aged lamb. With time, EAAs including isoleucine, lysine, threonine and valine, and non-essential AAs (non-EAAs) comprising arginine, aspartic acid, cysteine, histidine and proline, significantly increased in dry-aged lamb compared to the wet-aged equivalents (Table 7, 60 min, *p* < 0.05). A significant difference in FAAs observed between ageing treatments at 120 min of gastric digestion was not observed following the 120 min of pancreatic digestion (*p* > 0.05), probably due to the greater cleavage of protein fragments by pancreatic enzymes that resulted in similar levels of FAAs being released in both ageing treatments. 

When considering the FAA level between ageing treatments across the digestion process, three EAAs (isoleucine, threonine and valine) and most of the non-EAAs (except for hydroxyproline and arginine) were significantly (*p* < 0.05) higher in dry-aged lamb as compared to the wet-aged control. Therefore, based on the release of FAAs during gastric digestion, dry-aged lamb was more digestible than the wet-aged equivalent. The higher digestibility of dry-aged lamb products could also be attributed to the significantly (*p* < 0.05) higher yeast counts in the dry-aged sample compared to the control (Table 2). The presence of yeast in meat products has been associated with an increase in pH and the production of peptides, free fatty acids, FAAs and flavour compounds [52,57,58]. Thus, it is our assumption that a mild fermentation induced by yeast may have occurred during the dry-ageing process that could have contributed to the higher FAAs and smaller peptides in dry-aged lamb. Further studies are required to isolate and identify the yeast strain and to determine its role in the improvement of proteolysis and digestibility. 

#### 3.5.3. Protein Content in Hydrolysate

The protein content (protein fragments and peptides) in the final hydrolysates following 240-min simulated digestion was determined and no significant difference (*p* > 0.05) between the two ageing treatments was found (data not shown). Only 70% of the proteins were hydrolysed, with around 30% of protein hydrolysates remaining in the digests. This could be explained by the limitation of pancreatic proteases, which accounts for only 30–40% of protein hydrolysis occurring in human gastrointestinal digestion [55,59]. 

#### 3.5.4. Overall Relative Protein Digestibility (%)

The relative protein digestibility of dry-aged lamb products compared to the wet-aged control was determined by three methods. As shown in Table 8, both ageing regimes had similar digestibility regardless of the method used. Although the overall relative protein digestibility did not differ when the full 240 min of gastric and pancreatic digestions were considered together, it is important to note that from the analysis of the FAAs, we were able to determine that dry-aged meat had a higher gastric digestibility compared to wet-aged. This information is particularly relevant because the increased digestibility of meat at the upper gastrointestinal tract will minimise the chances of larger fragments of proteins reaching the lower gastrointestinal tract, where they may be fermented to produce metabolites that could contribute to bowel ailments in the elderly and those with compromised guts [60,61]. Thus, we recommend that a combination of assessment methods—such as the three used in the current study—be employed in order to fully determine the extent of the digestibility of meat.

## 4. Conclusions

This is the first time that in-bag dry-aged lamb was produced and a systematic study was performed to compare the product with its wet-aged equivalents in terms of meat quality, consumer acceptability, oxidative stability and digestibility considerations. The current findings demonstrated that the in-bag dry-ageing process can be used to produce highly acceptable lamb products with comparable meat quality and oxidative stability and with improved gastric digestibility compared to the wet-aged equivalents. Future studies focusing on determining the role of yeast in proteolysis during dry-ageing, and the improvement of gastric digestibility, will further validate the current findings. 

The outcomes also confirmed the niche nature of dry-aged meat compared to the equivalent wet-aged lamb and provided a formula for determining the yield of dry-aged lamb legs from its initial wet weight. 

A combination of SDS-PAGE, FAA and total protein measurement of digests enabled better assessment of meat digestibility than the use of each method alone. The use of FAA profiling in conjunction with SDS-PAGE will help us to better understand the biochemical processes during the digestion of meat products, particularly in the upper gastrointestinal tract, which is important when the bioaccessibility of the products is of interest.

## Figures and Tables

**Table 1:** Interview guide for the focus group of in-bag dry-aged lamb chops.

**(1)** **Introduction** Welcome and introduce participants. Explain research objectives and samples information. Explain the focus group procedure.
**(2)** **Consent of Voluntary Participation** Inform about recording equipment and how confidentiality will be protected. Obtain verbal consent from each of the participants.
**(3)** **Cooking and Eating** All the participants will be encouraged to observe the meat being cooked and note the aroma and general appearance of lamb chops. Lamb presented on a table with other food accompaniments. Participants invited to serve themselves one or more lamb chops.
**(4)** **Discussion on Why Like or Dislike Dry-Aged Lamb** Do you like or dislike the lamb chops? Why do you like or dislike the lamb chops? What do you like/dislike about the cooking process and aroma? What do you like/dislike about the aroma? What do you like/dislike about the appearance of cooked lamb chops? What do you like/dislike about the texture while eating? What do you like/dislike about taste? Do you like or dislike the flavour? Do you think this product has a strong sheep meat (mutton) flavour? What else do you like/dislike about the flavour?

**Table 2:** Effect of ageing treatments on the physico-chemical and microbial properties of lamb chops.

	W ^1^	BD ^1^	SED ^2^	*p*-Ageing
pH	5.92 ^a^	6.04 ^b^	0.02	<0.0001
% Moisture	73.25 ^a^	67.30 ^b^	0.37	<0.0001
% Crude fat	5.93	6.82	0.46	0.058
% Muscle protein	16.54 ^a^	20.22 ^b^	0.24	<0.0001
% Cook loss	27.79 ^a^	16.94 ^b^	0.67	<0.0001
% Total loss	28.48 ^a^	36.52 ^b^	0.56	<0.0001
**Meat Surface Microbial Count** (**Mean log cfu/g**)				
Aerobic bacteria	5.16 ^a^	2.68 ^b^	0.75	0.030
Lactic acid bacteria	2.64	n.d.	-	-
Moulds	n.d. ^3^	n.d.	-	-
Yeast	2.38 ^a^	3.75 ^b^	0.47	0.043
*Enterobacteriaceae*	2.36	n.d.	-	-
*Escherichia coli* (log MPN/g)	n.d.	n.d.	-	-

^1^ W and BD refer to wet-aged and in-bag dry-aged, respectively; ^2^ SED is the standard error of a difference between means; ^3^ n.d. refers to not detectable (under detection limit, <1.00 log cfu/g for moulds and *Enterobacteriaceae*, and <0.48 log MPN/g for *Escherichia coli*); Different superscript letters “a, b” within the same row indicate that results significantly differed from each other (*p* < 0.05).

**Table 3:** Effect of ageing treatments and muscle types on instrumental colour of lamb chops.

	W ^1^	BD ^1^	SED ^2^	*p*-Ageing	*p*-Muscle (Across Treatments)	*p*-Ageing (Across Muscles)
L*						
*SM* ^3^	44.01 ^ax^	42.57 ^b^	0.56	0.017	<0.0001	<0.0001
*BF* ^3^	44.52 ^ax^	42.66 ^b^		<0.0001		
*VL* ^3^	45.78 ^ay^	43.40 ^b^		<0.0001		
*RF* ^3^	45.70 ^ay^	43.63 ^b^		0.002		
*p*-muscle	0.002	0.202				
a*						
*SM*	14.92 ^axy^	13.84 ^bx^	0.52	0.041	<0.0001	<0.0001
*BF*	15.95 ^az^	14.40 ^bxy^		0.007		
*VL*	15.78 ^xz^	14.97 ^y^		0.092		
*RF*	14.39 ^ay^	12.43 ^bz^		0.001		
*p*-muscle	0.004	< 0.001				
b*						
*SM*	12.62 ^ax^	11.66 ^bx^	0.39	0.011	<0.0001	<0.0001
*BF*	13.59 ^ay^	12.32 ^bxy^		0.003		
*VL*	13.75 ^ay^	12.64 ^by^		0.003		
*RF*	13.13 ^ax^	11.17 ^bz^		<0.0001		
*p*-muscle	0.009	0.003				
Chroma						
*SM*	19.56 ^ax^	18.11 ^bx^	0.63	0.020	<0.0001	<0.0001
*BF*	20.96 ^ay^	18.96 ^bxy^		0.004		
*VL*	20.93 ^ay^	19.59 ^by^		0.024		
*RF*	19.49 ^ax^	16.73 ^bz^		<0.001		
*p*-muscle	0.009	< 0.001				
*Hue*						
*SM*	40.34 ^x^	40.20 ^x^	0.47	0.803	<0.0001	0.216
*BF*	40.47 ^x^	40.67 ^x^		0.611		
*VL*	41.11 ^ax^	40.25 ^bx^		0.017		
*RF*	42.49 ^y^	42.12 ^y^		0.501		
*p*-muscle	< 0.0001	< 0.001				

^1^ W and BD refer to wet-aged and in-bag dry-aged, respectively; ^2^ SED is the standard error of a difference between means; ^3^ *SM*, *BF*, *VL* and *RF* refer to *m. semimembranosus*, *m. biceps femoris*, *m. vastus lateralis* and *m. rectus femoris*, respectively; Different superscript letters “a, b” within the same row indicate that results significantly differed from each other (*p* < 0.05); Different superscript letters “x, y, z” within the same column indicate that results significantly differed from each other (*p* < 0.05).

**Table 4:** Effect of ageing treatments and muscle types on the texture profile analysis of the lamb chops.

	W ^1^	BD ^1^	SED ^2^	*p*-Ageing	*p*-Muscle (Across Treatments)	*p*-Ageing (Across Muscles)
Hardness (N)						
*SM* ^3^	21.14 ^a^	25.27 ^b^	1.66	0.015	0.366	<0.001
*BF* ^3^	22.53	26.50		0.067		
*VL* ^3^	22.48 ^a^	27.19 ^b^		0.004		
*RF* ^3^	22.17	24.45		0.120		
*p*-muscle	0.680	0.465				
Chewiness (N)						
*SM*	7.47	8.46	0.69	0.134	0.017	<0.001
*BF*	8.22 ^a^	9.89 ^b^		0.048		
*VL*	7.67 ^a^	9.46 ^b^		0.014		
*RF*	7.27	7.87		0.309		
*p*-muscle	0.414	0.051				
Springiness						
*SM*	0.62 ^xy^	0.63 ^x^	0.02	0.514	<0.0001	0.427
*BF*	0.65 ^x^	0.65 ^x^		0.979		
*VL*	0.61 ^y^	0.62 ^xy^		0.463		
*RF*	0.59 ^y^	0.60 ^y^		0.657		
*p*-muscle	0.012	0.010				
Cohesiveness						
*SM*	0.56 ^a^	0.53 ^b^	0.01	0.002	0.597	0.001
*BF*	0.55	0.54		0.360		
*VL*	0.55	0.54		0.497		
*RF*	0.55	0.53		0.057		
*p*-muscle	0.373	0.354				
Adhesiveness						
*SM*	−11.56	−18.13	4.10	0.229	0.054	0.335
*BF*	−9.18	−8.35		0.229		
*VL*	−10.93	−12.31		0.736		
*RF*	−7.08	−8.44		0.593		
*p*-muscle	0.439	0.127				
Resilience						
*SM*	0.23 ^a^	0.21 ^b^	0.01	0.017	0.284	0.048
*BF*	0.22	0.22		0.845		
*VL*	0.22	0.22		0.465		
*RF*	0.22	0.21		0.356		
*p*-muscle	0.123	0.443				

^1^ W and BD refer to wet-aged and in-bag dry-aged, respectively; ^2^ SED is the standard error of a difference between means; ^3^ *SM*, *BF*, *VL* and *RF* refer to *m. semimembranosus*, *m. biceps femoris*, *m. vastus lateralis* and *m. rectus femoris*, respectively; Different superscript letters “a, b” within the same row indicate that results significantly differed from each other (*p* < 0.05). Different superscript letters “x, y” within the same column indicate that results significantly differed from each other (*p* < 0.05).

**Table 5:** Effect of ageing treatments on the sensory quality of lamb chops.

	W ^1^	BD ^1^	SED ^2^	*p*-Ageing
Degree of Liking	6.75	6.68	0.19	0.682
% preferred ^3^	44.74	40.35		
Eating Quality Rating	3.14	3.10	0.13	0.750
% rating ^4^				0.670
Unsatisfactory as an everyday product	2.65	4.42		
Good everyday product	29.20	23.89		
Slightly better than an everyday product	31.86	37.17		
Almost a premium product	24.78	26.55		
A premium product	11.50	7.96		

^1^ W and BD refer to wet-aged and in-bag dry-aged, respectively; ^2^ SED is the standard error of a difference between means; ^3^ results were calculated as % consumers rated W or BD sample higher than its equivalent; ^4^ results were calculated as % consumers rated on the five-point scale eating quality groups of W and BD samples.

**Table 6:** Effect of ageing treatments on the oxidative changes in lipid and protein of lamb chops.

	W ^1^	BD ^1^	SED ^2^	*p*-Ageing
Protein Carbonyl (nmol/mg Protein)	2.20	2.31	0.08	0.151
TBARS ^3^ (mg MDA ^3^/kg Meat)	0.38 ^a^	1.30 ^b^	0.13	<0.0001
Fatty Acid Profile (mg/g dry Matter)				
10:0	0.18	0.17	0.02	0.631
12:0	0.35	0.33	0.03	0.539
14:0	2.56	2.44	0.22	0.580
14:1	0.09	0.09	0.01	0.490
15:0	0.27	0.25	0.02	0.390
16:0	12.71	12.44	0.72	0.706
16:1	1.00	1.01	0.07	0.879
17:0	1.10	1.07	0.05	0.542
17:1	0.38	0.37	0.02	0.490
18:0	10.89	10.60	0.57	0.606
18:1 (*n* ^3^—9, cis & trans)	18.37	18.22	1.06	0.887
18:2 (*n*—6, cis & trans)	2.00	2.02	0.07	0.715
18:3 (*n*—6)	0.21	0.21	0.02	0.848
18:3 (*n*—3)	1.31	1.29	0.05	0.713
20:0	0.19	0.20	0.01	0.272
20:2 (*n*—6)	0.15	0.16	0.01	0.429
20:6 (*n*—6)	0.23	0.23	0.01	0.873
20:5 (*n*—3)	0.69	0.70	0.02	0.801
22:0	0.27	0.27	0.01	0.900
24:0	0.20	0.20	0.01	0.946
22:6 (*n*—3)	0.36	0.35	0.01	0.725
UFAs ^3^	24.80	24.65	1.24	0.905
SFAs ^3^	28.72	27.96	1.47	0.609
MUFAs ^3^	19.84	19.68	1.14	0.890
*n*—3	2.36	2.34	0.05	0.756
*n*—6	2.59	2.62	0.08	0.734
PUFAs ^3^	4.95	4.96	0.12	0.928
% UFAs	46.40	46.92	0.47	0.266
% SFAs	53.60	53.08	0.47	0.266
% MUFAs	36.87	37.15	0.54	0.609
*% n*—3	4.56	4.64	0.22	0.721
*% n*—6	4.96	5.14	0.20	0.390
% PUFAs	9.52	9.77	0.40	0.534

^1^ W and BD refer to wet-aged and in-bag dry-aged, respectively; ^2^ SED is the standard error of a difference between means; ^3^ TBARS, MDA, UFAs, SFAs, MUFAs, PUFAs and *n* refer to thiobarbituric acid reactive substances, malondiadehyde, unsaturated fatty acids, saturated fatty acids, mono-unsaturated fatty acids, poly-unsaturated fatty acids and omega, respectively; Different superscript letters “a, b” within the same row indicate that results significantly differed from each other (*p* < 0.05).

**Table 7:** Effect of ageing treatments on the release of free amino acids (mg/g protein) of lamb chops at different digestion stages of the in vitro digestion process.

	0 Min			2 Min			10 min			60 Min			120 Min			240 Min			SED ^2^	*p*-Ageing (Across Digestion Time)
	W ^1^	BD ^1^	*p*-Ageing	W	BD	*p*-Ageing	W	BD	*p*-Ageing	W	BD	*p*-Ageing	W	BD	*p*-Ageing	W	BD	*p*-Ageing		
Essential amino acids																				
Histidine	0.36	0.46	0.252	0.35	0.41	0.202	0.37	0.40	0.481	0.42 ^a^	0.60 ^b^	0.021	0.38 ^a^	0.57 ^b^	0.001	0.73 ^a^	0.87 ^b^	0.034	0.06	<0.0001
Isoleucine	1.24 ^a^	1.57 ^b^	0.018	0.87	0.94	0.570	0.87	0.92	0.458	0.97 ^a^	1.21 ^b^	0.049	1.00 ^a^	1.31 ^b^	0.005	1.80	2.01	0.153	0.10	<0.0001
Leucine	1.01	0.98	0.907	1.49	1.60	0.574	1.51	1.59	0.507	1.77	2.18	0.075	1.89 ^a^	2.49 ^b^	0.005	12.13	12.75	0.591	0.50	0.150
Lysine	1.01 ^a^	1.19 ^b^	0.001	1.27	1.39	0.528	1.46	1.59	0.463	1.42 ^a^	2.01 ^b^	0.021	1.37 ^a^	2.10 ^b^	0.002	13.56	13.36	0.922	0.88	0.505
Methionine	0.66	0.72	0.491	0.66	0.71	0.571	0.59	0.59	0.996	0.73	0.82	0.410	0.79 ^a^	0.97 ^b^	0.030	1.89	2.05	0.438	0.11	0.063
Phenylalanine	1.60	2.00	0.144	1.00	1.03	0.669	0.99	1.01	0.632	1.25	1.38	0.180	1.46 ^a^	1.70 ^b^	0.025	10.41	10.91	0.632	0.44	0.218
Threonine	1.34	1.73	0.153	0.56	0.63	0.285	0.63	0.77	0.305	0.67 ^a^	0.98 ^b^	0.045	0.65 ^a^	1.01 ^b^	0.007	0.82 ^a^	1.05 ^b^	0.004	0.14	<0.0001
Tryptophan	0.24	0.30	0.238	0.53	0.55	0.424	0.55	0.56	0.704	0.63	0.68	0.125	0.67 ^a^	0.75 ^b^	0.011	2.55	2.58	0.898	0.11	0.372
Valine	1.01	1.27	0.087	1.16	1.26	0.518	1.23	1.29	0.532	1.32 ^a^	1.70 ^b^	0.038	1.36 ^a^	1.85 ^b^	0.005	1.92 ^a^	2.34 ^b^	0.028	0.14	<0.0001
Non-essential amino acids																				
Alanine	1.97	2.21	0.547	3.25	3.57	0.405	3.38	3.51	0.771	3.78	4.36	0.242	3.92 ^a^	4.93 ^b^	0.034	3.91	4.60	0.101	0.39	0.004
Arginine	1.35	1.63	0.152	1.04	1.16	0.380	1.02	1.08	0.599	1.25 ^a^	1.49 ^b^	0.034	1.40	1.64	0.155	20.02	20.12	0.963	0.91	0.643
Asparagine	0.45	0.59	0.124	0.46	0.53	0.227	0.52	0.55	0.585	0.82	0.82	0.768	1.24 ^a^	1.73 ^a^	0.004	1.46 ^a^	1.84 ^b^	0.008	0.11	<0.0001
Aspartic acid	0.35 ^a^	0.64 ^b^	0.043	0.49	0.52	0.688	0.70	0.76	0.446	0.60 ^a^	1.00 ^b^	0.004	0.63 ^a^	1.15 ^b^	0.000	0.67 ^a^	1.11 ^b^	0.000	0.09	<0.0001
Cysteine	0.04	0.04	0.236	0.69 ^a^	0.83 ^b^	0.014	0.85	0.98	0.412	0.91 ^a^	1.19 ^b^	0.046	0.91 ^a^	1.30 ^b^	0.002	1.18	1.38	0.233	0.11	<0.0001
Glutamic acid	0.93	1.16	0.354	1.13	1.24	0.534	1.25	1.17	0.630	1.36	1.59	0.139	1.47 ^a^	1.81 ^b^	0.007	1.36 ^a^	1.69 ^b^	0.044	0.16	0.005
Glutamine	3.67	4.18	0.447	2.97	3.26	0.567	2.94	3.22	0.672	3.68	4.14	0.367	3.43	4.58	0.056	3.68	4.50	0.258	0.59	0.017
Glycine	1.19	1.28	0.603	2.80	2.92	0.659	2.71	2.91	0.254	2.94	3.19	0.232	2.92 ^a^	3.50 ^b^	0.008	3.29	3.78	0.054	0.20	0.001
Hydroxyproline	0.11	0.11	0.847	0.04	0.05	0.302	0.04	0.04	0.580	0.06	0.05	0.218	0.05	0.06	0.094	0.05	0.06	0.067	0.01	0.051
Proline	0.50 ^a^	0.71 ^b^	0.045	0.42	0.45	0.534	0.47	0.51	0.468	0.50 ^a^	0.65 ^b^	0.031	0.60 ^a^	0.85 ^b^	0.007	0.50 ^a^	0.67 ^b^	0.014	0.06	<0.0001
Serine	1.20	1.46	0.259	1.38	1.50	0.530	1.50	1.56	0.668	1.62	2.04	0.055	1.70 ^a^	2.24 ^b^	0.003	1.69 ^a^	2.17 ^b^	0.008	0.17	<0.0001
Tyrosine	0.93	1.13	0.361	0.93	0.99	0.603	0.93	0.98	0.384	1.11	1.29	0.129	1.15 ^a^	1.43 ^b^	0.008	8.63	9.01	0.657	0.37	0.203
EAAs ^3^	8.48	10.23	0.092	7.88	8.52	0.498	8.20	8.72	0.460	9.23	11.56	0.044	9.56	12.75	0.005	45.81	47.92	0.682	2.24	0.063
% EAAs	40.43	40.65	0.895	33.53	33.39	0.874	33.56	33.73	0.877	33.46	34.55	0.484	32.98	33.57	0.569	49.51	48.53	0.258	1.15	0.790
Total AAs ^3^	21.17	25.36	0.211	23.48	25.54	0.458	24.51	25.99	0.547	27.82	33.37	0.052	28.99 ^a^	37.96 ^b^	0.003	92.25	98.84	0.499	4.59	0.014

^1^ W and BD refer to wet-aged and in-bag dry-aged, respectively; ^2^ SED is the standard error of a difference between means; ^3^ AAs and EAAs refer to amino acids and essential amino acids; Different superscript letters “a, b” within the same row indicate that results significantly differed from each other (*p* < 0.05).

**Table 8:** Overall relative digestibility (%) of lamb chops following 240-min in vitro digestion.

	W ^1^	BD ^1^	*p*-Ageing	SED ^2^
Relative digestibility _SDS-PAGE_	63.96	65.24	0.776	4.36
Relative digestibility _FAAs_	7.11	7.35	0.802	0.92
Relative digestibility _protein content_	70.18	70.75	0.570	0.95

^1^ W and BD refer to wet-aged and in-bag dry-aged, respectively; ^2^ SED is the standard error of a difference between means.

## Data Availability

The data presented in this study are available on request from the corresponding author.
